# A large Venous-Arterial PCO_2_ Is Associated with Poor Outcomes in Surgical Patients

**DOI:** 10.1155/2011/759792

**Published:** 2011-10-05

**Authors:** João M. Silva, Amanda M. Ribas R. Oliveira, Juliano Lopes Segura, Marcel Henrique Ribeiro, Carolina Nacevicius Sposito, Diogo O. Toledo, Ederlon Rezende, Luiz M. Sá Malbouisson

**Affiliations:** ^1^Anestesiologia e Cuidados Intensivos, Hospital do Servidor Público Estadual, 04039-901 São Paulo, SP, Brazil; ^2^De Terapia Intensiva, Hospital do Servidor Público Estadual, 04039-901 São Paulo, SP, Brazil; ^3^Anestesiologia, Hospital do Servidor Público Estadual, 04039-901 São Paulo, SP, Brazil; ^4^De Terapia Intensiva, Hospital das Clínicas, 05403-900 São Paulo, SP, Brazil

## Abstract

*Background*. This study evaluated whether large venous-arterial CO_2_ gap (PCO_2_ gap) preoperatively is associated to poor outcome. *Method*. Prospective study which included adult high-risk surgical patients. The patients were pooled into two groups: wide [P(v-a)CO_2_] versus narrow [P(v-a)CO_2_]. In order to determine the best value to discriminate hospital mortality, it was applied a ROC (receiver operating characteristic) curve for the [P(v-a)CO_2_] values collected preoperatively, and the most accurate value was chosen as cut-off to define the groups. *Results*. The study included 66 patients. The [P(v-a)CO_2_] value preoperatively that best discriminated hospital mortality was 5.0 mmHg, area = 0.73. Preoperative patients with [P(v-a)CO_2_] more than 5.0 mmHg presented a higher hospital mortality (36.4% versus 4.5% *P* = 0.004), higher prevalence of circulatory shock (56.8% versus 22.7% *P* = 0.01) and acute renal failure postoperatively (27.3% versus 4.5% *P* = 0.02), and longer hospital length of stays 20.0 (14.0–30.0) versus 13.5 (9.0–25.0) days *P* = 0.01. *Conclusions*. The PCO_2_ gap values more than 5.0 mmHg preoperatively were associated with worse postoperatively outcome.

## 1. Introduction

Although every year millions of surgeries are performed all around the world, few patients that undergo major surgeries are judged to be of high risk for postoperative complications and death [1]. An observational study reported that only 12.5% of all surgical procedures are considered high risk, but over 80% of the deaths which occurred were in the high-risk group [1]. Despite the high mortality rate, less than 15% of those patients are admitted to intensive care units (ICU) [1], which shows that the individual risk may easily be underestimated and that high-risk patients may not be recognized.

The mechanisms by which inflammatory response and tissue hypoperfusion occur in major surgery are still not entirely clear, but evidence indicates that oxygen requirements increase significantly as a result of the injury and metabolic response to the trauma caused by the surgery. However, very often high-risk patients are not able to increase their cardiac output and thus the oxygen delivery [2]. Therefore, those patients will more likely develop tissue hypoperfusion and severe systemic inflammatory response and death due to the organ dysfunction [3]. 

Surgical patients with a high risk for complications admitted to an ICU frequently die as a primary or secondary consequence of hypoperfusion-related organ dysfunction or severe infections [4, 5]. However, predictors of death caused by tissue hypoperfusion are still not studied very much in high-risk surgery patients. The [P(v-a)CO_2_] has been reversely correlated to cardiac output in surgical patients [6–8]. In a recent review, Lamia et al. [9] showed that PCO_2_ gap is a blood flow marker to remove total CO_2_ produced by the peripheral tissue. Cuschieri et al. [10] have found a reverse correlation between [P(cv-a)CO_2_] and cardiac index with a central venous blood sample and suggested that, for this purpose, a sample of central venous blood could be used instead of blood from the pulmonary artery.

In the literature, we find only very few prospective clinical studies with surgical patients, which have evaluated the real impact from [P(v-a)CO_2_] as complications and death preoperative marker in this population. Therefore, the objective of this study was to evaluate the role of PCO_2_ gap as a predictor for complications and death in high-risk surgical patients.

## 2. Methods

The study was performed in a tertiary hospital. It was approved by the local Ethics and Research Committees, and a written postinformed consent was obtained from each patient or legal responsible. The study design was a post hoc analysis applying a cut point derived from the data, with the following inclusion criteria: (1) patients aged 18 years or older and (2) surgeries which required a central venous catheter in intraoperative and intensive care in postoperative phase. Patients were selected during the preanesthetic evaluation. High-risk surgeries were patients undergoing surgeries with requested ICU postoperative stay and at least one of the following:

- severe cardiorespiratory comorbidities (coronary insufficiency, chronic obstructive pulmonary disease, previous stroke),
- surgery scheduled for neoplasm resection (esophagectomy, total gastrectomy) longer than 8 hours,
- above 70 years-old, with evidence of physiological reserve impairment involving at least one vital organ,
- acute renal failure (blood urea nitrogen > 100 mg/dL or creatinine > 3 mg/dL),
- advanced vascular disease, or aortal involvement,
- massive acute intraoperative blood loss predicted,
- severe nutritional disorders patients.

Patients undergoing palliative surgery, with low life expectancy, liver failure (Child B or C), patients with a functional class IV heart failure or an echocardiogram-measured ejection fraction of less than 30%, and those who did not accept participating in the study were excluded. The patients with a functional class IV heart failure or ejection fraction of less than 30% could present flow alteration due to underlying disease, which is not related to surgery procedure. 

At the time of the inclusion, the Multiple-Organ Dysfunction Syndrome (MODS) [11] and Acute Physiology And Chronic Health Evaluation (APACHE II) [12] scores were also evaluated by taking the worst values of their variables. An echocardiogram, which is an integral part of preoperative evaluation of high-risk surgical patients in our institution, was performed to measure the ejection fraction and the presence or absence of systolic or diastolic ventricular dysfunction, considering the increasingly clear literature data on their similarities regarding mortality and morbidity [13–16].

At the day of the surgery, patients were equipped with central venous (positioned with the tip within the superior vena cava) and arterial catheters. A chest X-ray confirmed central venous catheter position. Before anesthesia induction, in intraoperatively and postoperatively central venous and arterial blood samples were collected to measure [P(v-a)CO_2_]. 

The surgical team decided on which therapy should be used during the intraoperative period. In the postoperative phase, the intensive care physician's goal was to improve the perfusion parameters according to standard in local institution; he had no knowledge about preoperative [P(v-a)CO_2_].

The primary endpoint was hospital mortality; all patients were therefore monitored until they were discharged from hospital. The secondary endpoint was to check for the presence of an organ dysfunction, shock (need of vasoactive drugs for over 1 hour, despite volemic resuscitation), acute pulmonary dysfunction (PaO_2_/FiO_2_ ratio < 200), renal failure (increase of creatinine by 50% or urinary output of less than 400 mL in 24 hours), confusion (change in behavior, memory lapses, or psychomotor agitation), and platelet dysfunction (platelets reduced by 30% compared to the basal value) in up to 24 hours in the postoperative period. Diagnosis of postoperative infection was based on international consensus guidelines and/or use of antibiotic [17]. The infections incidence during ICU length of stay and the time of mechanical ventilation were verified, besides the hospital length of stay.

 Based on the best [P(v-a)CO_2_] value which discriminated hospital mortality, the patients were allocated in two groups: narrow [P(v-a)CO_2_] (group 1) and wide [P(v-a)CO_2_] (group 2).

### 2.1. Statistical Analysis

Initially, we described the demographic, clinical, and physiological characteristics from patients that were included in the study. Frequencies were calculated to describe categorical variables. Quantitative variables were described by using measures of central tendency and dispersion.

The statistical method to be used in the assessment of each variable was chosen based on its distribution. Categorical variables were analyzed through the Chi-square test, and continuous variables through the mean with the Student's *t*-test for normal distribution and continuous variables with irregular distribution were analyzed by means of the Mann-Whitney Test. *P*  values < 0.05 (two-tailed) were considered significant. ROC curves were used to test the discrimination power of the groups in predicting clinical evolution (the capacity of correctly classifying survivors and nonsurvivors) and to determine the best cut-off value of tissue perfusion in connection with hospital mortality. SPSS 13.0 was used to analyze those calculations. Group 01 patients were compared to those of group 02. Box plots were created to compare survivors and nonsurvivors in [P(v-a)CO_2_] in different time points through Mann-Whitney test.

To determine the risk of hospital death we developed a multivariable Cox proportional hazard model in the population. Variables were included in the model if they reached a significance level of *P* < 0.05 in univariate analysis or were clinically relevant. Mortality estimative curve was created using the Cox method and compared by Cox regression.

## 3. Results

Over a period of 6 months, the study included 66 patients, of which 37 males and 29 females, with an average age of 65.6 years old. Elective surgeries were more frequent (Table 1).

During surgery, 50% of patients received blood transfusions, 32.5% vasoactive drugs, and 54.5% of them had complications, where circulatory shock was most prevalent (Table 2). 

**Figure 1:**
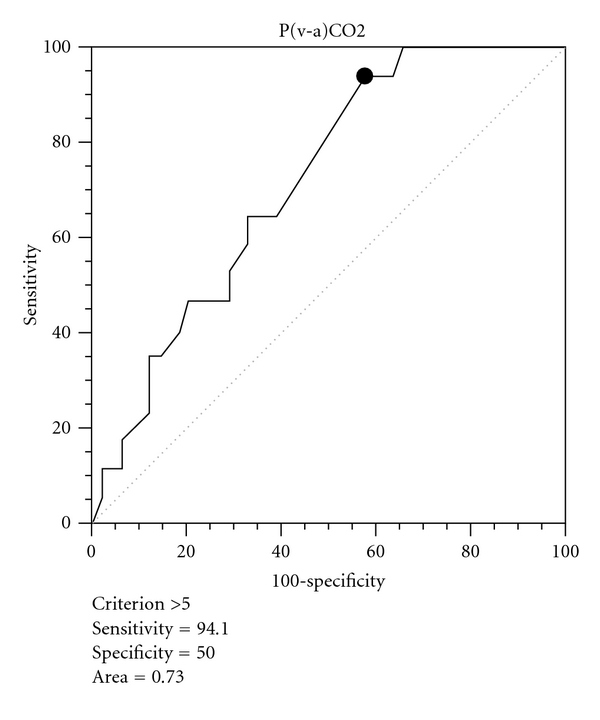
Roc curve from [P(v-a)CO_2_] and hospital mortality.

 A ROC curve was created in order to establish the best [P(v-a)CO_2_] value, which discriminated hospital mortality in this population, where [P(v-a)CO_2_] = 5.0 mmHg was the cut-off between narrow [P(v-a)CO_2_] and wide [P(v-a)CO_2_], presenting sensitivity of 93.3% and specificity of 50.2%, ROC area of 0.73, *P*(area = 0.5) = 0.0006, 95% CI = 0.61 to 0.84 (Figure 1). Moreover, comparative ROC curves from lactate, BE and ScvO_2_ can demonstrate that [P(v-a)CO_2_] had the greatest AUC, respectively, 0.53 (0.41 to 0.67), 0.56 (0.43 to 0.69), and 0.71 (0.59 to 0.82).

**Figure 2:**
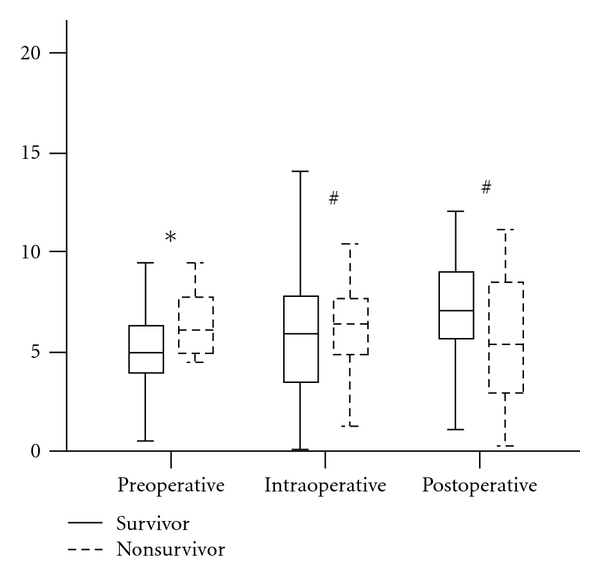
Box plot between [P(v-a)CO_2_] value from survivors and nonsurvivors (*P < 0.05; ^#^ *P* > 0.05).

A Box plot graphic has compared survivors and non survivors in pre-, intra-, and postoperatively; it could show that only the preoperatively period presented statistical significance between them (Figure 2).

Thus, when comparing the groups with narrow [P(v-a)CO_2_] and wide [P(v-a)CO_2_], although they present the same pre- and intraoperative demographic and clinical data, except for a lower ScvO_2_ and a higher ventricular dysfunction in patients with [P(v-a)CO_2_] ≥ 5.0 mmHg, we find that patients with [P(v-a)CO_2_] ≥ 5.0 mmHg had a higher hospital mortality and a higher incidence of complications in the postoperative period, mainly circulatory shock, renal failure, and ICU infection. (Tables 1, 2, 3, and 4)

**Figure 3:**
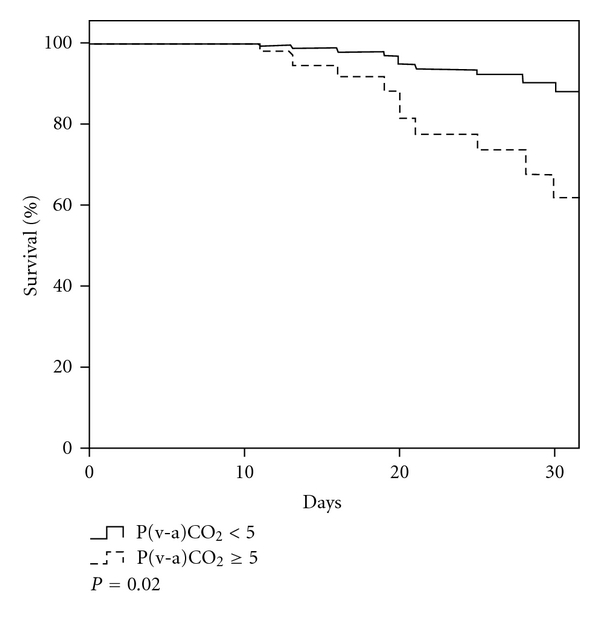
Kaplan Meier curve.

When patients with [P(v-a)CO_2_] < 5.0 mmHg and [P(v-a)CO_2_] ≥ 5.0 mmHg were compared, the group with [P(v-a)CO_2_] ≥ 5.0 mmHg had a lower survival probability, even when it was adjusted by MODS, ScvO2, ventricular dysfunction heart. HR = 2.07 IC95% 1.14–3.77 (Figure 3).

## 4. Discussion

Despite the large quantity of resources that are directed towards assessing perioperative risk and of cardiovascular complications, this study shows that a simple analysis of PCO_2_ gap ≥ 5.0 mmHg in the preoperative period is an important marker for postoperative complications, mainly shock, renal failure, infection, increased length hospital stay, and hospital mortality. Currently, few parameters are used at bed side to assess tissue hypoxia, such as urinary output, base differences, and blood lactate, but those parameters show that hypoperfusion is already installed and may be late to guide the onset of a hemodynamic resuscitation [18]. However, in this study, [P(v-a)CO_2_] has proven to be an early parameter to identify worse outcome in surgery patients.

The worse outcome of these patients can be explained by previous reports that have suggested the PCO_2_ gap as a marker for tissue hypoxia [19, 20]. In an animal model for acute hemorrhage, Van der Linden et al. [21] found a significant correlation between blood lactate and PCO_2_ gap. A progressive increase of [P(v-a)CO_2_] was observed during the dependence period of oxygen consumption and delivery (VO_2_/DO_2_) in another experimental model with progressive flow reduction [22]. High [P(v-a)CO_2_] values have been reported during cardiac arrest and cardiopulmonary resuscitation [19, 23]. However, in all those studies, tissue hypoxia was secondary to the reduction of blood flow. 

Circulatory failure is followed by tissue CO_2_ accumulation. Tissue CO_2_ increases as a consequence of an accumulation of the carbon dioxide produced by the aerobic metabolism and by the tissue bicarbonate buffer effect, which is necessary to correct the excess of hydrogen ions during the anaerobic metabolism. When the cell concentration of bicarbonate ion (HCO_3_^−^) and hydrogen ion (H^+^) rises, HCO_3_^−^ diffuses out of red blood cells into the plasma, but H^+^ cannot diffuse easily because the cell membrane is relatively impermeable to cations. Some of H^+^ liberated are bound to hemoglobin (Hb). This reaction occurs because reduced Hb is a better acceptor of H^+^ than the oxygenated Hb. In the peripheral blood the loading of CO_2_ is facilitated by the presence of reduced Hb (Haldane effect) [9]. 

Low blood flow can result in tissue hypercapnia. Under anaerobic metabolism conditions, it is expected that the production of CO_2_ (VCO_2_) decreases less than the consumption of O_2_ (VO_2_). In other words, the VCO_2_/VO_2_ ratio (respiratory quotient) should increase. According to the Fick equation, a low blood flow may result in an increase of [P(v-a)CO_2_], even without any additional production of CO_2_. This can be explained by the CO_2_-stagnation phenomenon [24] due to the slower microcirculation blood flow, which is larger than normal CO_2_ production; hypercapnia tends to be generated in the venous circulation. 

Therefore, [P(v-a)CO_2_] is proportional to CO_2_ production and inversely related to cardiac output (Fick equation). Anaerobic CO_2_ production occurs when tissue hypoxia is present, mainly to protect bicarbonate ions against excess protons, produced as a secondary consequence of adenosine triphosphate hydrolysis [25].

In this respect, an increased [P(v-a)CO_2_] value has been reported in patients with low cardiac output and no global tissue hypoxia, as demonstrated by the normal lactate levels that were found [26]. This emphasizes the lack of specificity of [P(v-a)CO_2_] in detecting tissue hypoxia. In this study, we observed that even patients with inadequate [P(v-a)CO_2_] values presented normal lactate or base difference values. Serum lactate has been traditionally accepted like an indicator of anaerobic metabolism and of tissue hypoxia, but it has to be emphasized that, under normal conditions, the liver is capable of increasing the metabolism of the produced lactate; this means that, in hypoxia and anaerobic metabolism situations, a few hours may elapse between the onset of the phenomenon and the detection of elevated lactate levels in the blood [27], which explains why the lactate may not be as early as of the [P(v-a)CO_2_].

Otherwise, a normal [P(v-a)CO_2_] value may be associated with global tissue hypoxia in normal or hyperdynamic states, which was demonstrated by some authors [28]. This fact explains why an elevated venous flow is sufficient to clear the CO_2_ that is produced by the hypoxic cell, even if the production of CO_2_ is higher than normal due to the anaerobiosis that generates CO_2_ [24]. Indeed, Vallet and colleagues evidenced that PCO_2_ gap increased during low blood flow-induced tissue hypoxia (ischemic hypoxia) while it remained unchanged during hypoxemia-induced hypoxia (hypoxic hypoxia) [29]. We emphasize once again the poor sensitivity of [P(v-a)CO_2_] to detect tissue hypoxia in some cases. However, in this study, patients with an inadequate [P(v-a)CO_2_] had a lower ScvO_2_, which may be explained by the fact that ScvO_2_ is influenced by different variables [30], such as low blood flow; this might be occurring with the sample in question. This hypothesis is substantiated by a higher percentage of ventricular dysfunction in this group.

The prevalence of ventricular dysfunction in this study was evaluated, considering both the systolic and diastolic ventricular dysfunction, since their evolution as for morbidity and mortality is similar [13–16]. A greater prevalence of ventricular dysfunction was found in the inadequate [P(v-a)CO_2_] group. The EF—close to 65% in both groups—reflects a predominant diastolic dysfunction, which substantiates current data, which present diastolic dysfunction as a more relevant prognosis predictor than EF per se. [31–34]. 

However, Vallée et al. [35] found that a ScvO_2_ of over 70% in septic patients was not enough to reach adequate resuscitation, and that [P(v-a)CO_2_] ≥6.0 mmHg could be helpful to identify patients that were not adequately resuscitated. Mekontso-Dessap et al. [24] demonstrated that, under conditions of tissue hypoperfusion, defined as a blood lactate level of >2.0 mmol/L, a PvCO_2_-PaCO_2_/Ca-vO_2_ ratio of >1.4 was the best predictor of hyperlactatemia and a good prognostic index.

Thus, it is important to detect early a risk factor for low oxygen delivery due to low blood flow evaluated by PCO_2_ gap ≥5.0 mmHg in surgery patients, because changes in clinical management may be made in order to avoid an unfavorable outcome in this population. Several clinical studies have documented that and aggressive early resuscitation using defined protocols improve the patient's outcome [36, 37]. These studies used therapy strategies to increase the cardiac function and keep organ perfusion, which reduced the ICU length of stay, accelerated the recovery of the gastrointestinal function, and reduced the mortality rate in high-risk surgery patients [38]. Therefore, [P(v-a)CO_2_] could help identify patients that require such optimizations in the preoperative phase and specific measures taken in the preoperative or intraoperative period to ensure that the increase of blood flow could bring benefits to the postoperative period and improve the prognosis. 

However, in this study, the [P(v-a)CO_2_] value that best discriminated mortality was lower than of those described previously in the literature. This can be probably explained by the fact that those studies discussed more severe patients with septic conditions that required high oxygen delivery, contrary to what was shown in the study in question, since their patients were in the preoperative period of elective surgeries, with low APACHE II values, most of them in ASA II physical condition. In addition, values of the inadequate [P(v-a)CO_2_] found in this evaluation may eventually mean that the patients were not well enough to undergo major surgeries, but tolerable for basal activities, which are not comparable to the stress experienced in a surgical procedure. 

Another aspect that has to be discussed is related to the fact that inadequate [P(v-a)CO_2_] was found only in the preoperative and not in the intraoperative phase; it is important to highlight that in the intraoperative period some variables may interfere in the measurement of [P(v-a)CO_2_], such as the anesthesia and hypothermia that might occur during surgery; they may reduce cell respiration and, thus, CO_2 _production. Hypothermia reduces metabolism by 7% per grade, with reduction of ATP formation and reduction of cellular oxygen and cerebral glucose requirements, in addition, decrease in CO_2_ production and O_2_ consumption [39].

Other aspects can bring impact to change in [P(v-a)CO_2_], due to CO_2_ dissociation curve, which is curvilinear more than oxygen dissociation curve, and temperature, hematocrit, oxygen saturation, and pH influence the PCO_2_/CO_2_ content relationship an example is showed when metabolic acidosis worsens; the relationship is shifted to the right; it means that the same CO_2_ content value can determinate superior PCO_2_ value [9].

Besides, although in most studies that evaluated [P(v-a)CO_2_] patients were monitored by means of a pulmonary artery catheter [24], absence of this tool in this study does not limit the results that were found. By the other hand, the gastric tonometry has also been utilized as carbon dioxide monitoring [40], but this method assesses the regional tissue dysoxia, differently of the proposal from present study which has aimed to measure the systemically problem. Therefore, since the study's objective does not require the measures that are evaluated by these devices, this way can be cheaper. 

The complications, except infection, were evaluated in 24 hours only, which makes a limitation, but the length of ICU stay of all patients was short, that is, median 3.0 days.

The sample sizes as well as the observational character of this study are limiting factors. Future researches are needed to validate this finding.

Therefore, patients who underwent major surgeries and that—in the preoperative period—presented a PCO_2_ gap ≥5 mmHg had a worse prognosis in the postoperative. This marker may turn out to be important to stratify risk and suggests a useful additional tool for perioperative management.

##  Conflict of Interests

The authors declare that there is no conflict of interests.

##  Authors' Contribution

J. M. S. Jr. conceived of this study, participated in the design of the study, performed the statistical analysis, and drafted the paper. A. M. R. R. Oliveira participated in the design of the study, performed the collection of data and the statistical analysis, and drafted the paper. J. L. Segura, M. H. Ribeiro, and C. N. Sposito performed the collection of data. D. O. Toledo, E. Rezende, and L. M. S. Malbouisson helped in revising of the draft, the paper and helped in the final revision of writing the paper. All authors read and approved the final paper.

## Figures and Tables

**Table 1:** Patient characteristics and comparison between patients with [P(v-a) CO_2_] < 5.0 and [P(v-a)CO_2_] ≥ 5.0.

Variables	All patients (*n* = 66)	[P(v-a)CO_2_] < 5.0 mmHg (*n* = 22)	[P(v-a)CO_2_] ≥ 5.0 mmHg (*n* = 44)	*P*
Age	65.6 ± 12.2	64.6 ± 13.5	66.0 ± 11.7	0.67
Males (%)	56.1	50.0	59.1	0.48
APACHE II	16.9 ± 5.6	15.8 ± 5.1	17.4 ± 5.8	0.29
MODS	3 (1.0–4.0)	3.0 (1.0–4.0)	3.5 (1.0–4.5)	0.46
ASA (%)				0.65
I	6.6	9.5	5.0	
II	68.9	71.4	67.5	
III	24.6	19.0	27.5	
Elective surgery (%)	95.5	95.5	95.5	1.00
Emergency surgery (%)	4.5	4.5	4.5	1.00
Gastrointestinal surgery (%)	78.8	81.8	73.3	0.38
[P (v-a) Co_2_] (mmHg)	5.4 ± 2.0	3.3 ± 1.2	6.5 ± 1.4	0.00
Lactate (mmol/L)	1.5 ± 0.8	1.4 ± 0.6	1.6 ± 0.9	0.35
Base excess (mmol/L)	−0.7 (−3.0–0.6)	−1.6 (−3.4–0.7)	−0.5 (−2.4–0.6)	0.35
ScvO_2_ (%)	74.1 ± 7.6	77.0 ± 6.2	72.7 ± 7.9	0.03
Ventricular dysfunction (%)	77.3	54.5	88.6	0.002
Ejection fraction (%)	63.7 ± 9.5	63.5 ± 9.9	63.9 ± 92	0.93
Hemoglobin (g/dL)	11.4 ± 1.7	11.3 ± 1.7	11.5 ± 1.7	0.70
Glucose (mg/dL)	120.0 ± 54.2	120.7 ± 61.7	120.3 ± 50.8	0.98

ScvO_2_-central venous oxygen saturation; ASA: American Society of Anesthesiologists; values between brackets represent the median and percentile 25–75%.

**Table 2:** Patient characteristics and comparison between patients with [P(v-a)CO_2_] < 5.0 and [P(v-a)CO_2_] ≥ 5.0 in intraoperative.

Variables	All patients (*n* = 66)	[P(v-a)CO_2_] < 5.0 mmHg (*n* = 22)	[P(v-a)CO_2_] ≥ 5.0 mmHg (*n* = 44)	*P*
Transfusion (%)	50.0	40.9	54.5	0.29
Crystalloids (mL)	7000 (4625–8875)	5500 (4500–8650)	7000 (5500–8875)	0.44
Colloids (mL)	1000 (500–1000)	500 (500–1250)	1000 (500–1000)	0.26
Fluid balance (ml)	800 (175–1420)	400 (−250–875)	800 (262–1750)	0.13
Vasopressors (%)	32.5	23.5	40.5	0.27
Length of surgery (hours)	6.9 ± 2.4	7.0 ± 3.1	6.8 ± 1.9	0.83

Values between brackets represent the median and percentile 25–75%.

**Table 3:** Patient characteristics and comparison between patients with adequate [P(v-a)CO_2_] < 5.0 and [P(v-a)CO_2_] ≥ 5.0 in postoperative.

Variables	All patients (*n* = 66)	[P(v-a)CO_2_] < 5.0 mmHg (*n* = 22)	[P(v-a)CO_2_] ≥ 5.0 mmHg (*n* = 44)	*P*
Transfusion (%)	18.2	18.2	18.2	1.00
Crystalloids (mL)	1900 (1000–2500)	1500 (1000–2875)	2000 (1000–2500)	0.77
Colloids (mL)	500 (500–875)	500 (500–750)	500 (500–1000)	1.00
Duration of mechanical ventilation (hours)	24 (12–24)	12 (12–24)	24 (12–30)	0.07

Values between brackets represent the median and percentile 25–75%.

**Table 4:** Outcomes.

Variables	All patients (*n* = 66)	[P(v-a)CO_2_] < 5.0 mmHg (*n* = 22)	[P(v-a)CO_2_] ≥ 5.0 mmHg (*n* = 44)	RR	*P*
Postoperative complications (%)	54.5	40.9	61.4	1.73	0.09
Shock	45.5	22.7	56.8	2.83	0.01
ARF	19.7	4.5	27.3	5.15	0.02
Platelet dysfunction	19.7	13.6	22.7	1.55	0.38
Infection	16.7	4.5	22.7	4.20	0.05
Acute pulmonary dysfunction	10.6	9.1	11.4	1.18	0.77
Confusional state	7.6	0.0	11.4	0.64	0.10
Length of ICU stay (days)	3.0 (2.0–4.0)	2.5 (1.0–4.0)	3.0 (2.0–4.5)		0.12
Hospital length of stay (days)	20.0 (12.5–27.5)	13.5 (9.0–25.0)	20.0 (14.0–30.0)		0.01
Hospital mortality (%)	25.8	4.5	36.4	2.10	0.004

ARF: acute renal failure; ICU: intensive care unit; values between brackets represent the median and percentile 25–75%; RR: relative risk.
